# 2-Bromo­pyridine-3-carboxylic acid

**DOI:** 10.1107/S1600536810003314

**Published:** 2010-01-30

**Authors:** R. Alan Howie, Raoni S. Gonçalves, Marcus V. N. de Souza, Edward R. T. Tiekink, James L. Wardell

**Affiliations:** a Department of Chemistry, University of Aberdeen, Old Aberdeen AB15 5NY, Scotland; b Instituto de Tecnologia em Farmacos, Fundação Oswaldo Cruz (FIOCRUZ), Far-Manguinhos, Rua Sizenando Nabuco, 100, Manguinhos, 21041-250 Rio de Janeiro, RJ, Brazil; c Department of Chemistry, University of Malaya, 50603 Kuala Lumpur, Malaysia; d Centro de Desenvolvimento Tecnológico em Saúde (CDTS), Fundação Oswaldo Cruz (FIOCRUZ), Casa Amarela, Campus de Manguinhos, Av. Brasil 4365, 21040-900 Rio de Janeiro, RJ, Brazil

## Abstract

The carboxylic acid residue in the title compound, C_6_H_4_BrNO_2_, is twisted out of the plane of the other atoms, as indicated by the (Br)C—C—C—O_carbon­yl_ torsion angle of −20.1 (9)°. In the crystal, supra­molecular chains mediated by O—H⋯N hydrogen bonds are formed with base vector [201] and C—H⋯O inter­actions reinforce the packing.

## Related literature

For the biological activity of *N*-heterocylic compounds, see: de Souza (2005 ▶); Cunico *et al.* (2006 ▶). For related structures, see: Wright & King (1953 ▶); Kutoglu & Scheringer (1983 ▶); de Souza *et al.* (2005 ▶); Kaiser *et al.* (2009 ▶). For the synthesis, see: Bradlow & van der Werf (1949 ▶).
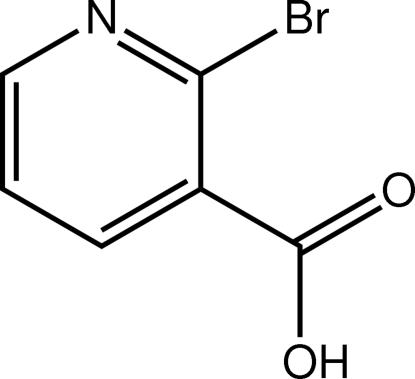


## Experimental

### 

#### Crystal data

C_6_H_4_BrNO_2_
*M* *_r_* = 202.01
Monoclinic, 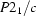

*a* = 3.9286 (3) Å
*b* = 12.9737 (9) Å
*c* = 12.8570 (8) Å
β = 96.695 (4)°
*V* = 650.83 (8) Å^3^
*Z* = 4
Mo *K*α radiation
μ = 6.24 mm^−1^
*T* = 120 K
0.10 × 0.09 × 0.08 mm

#### Data collection

Nonius KappaCCD area-detector diffractometer
Absorption correction: multi-scan (*SADABS*; Sheldrick, 2007 ▶) *T*_min_ = 0.453, *T*_max_ = 0.607
7699 measured reflections
1147 independent reflections
882 reflections with *I* > 2σ(*I*)
*R*_int_ = 0.070

#### Refinement

*R*[*F* ^2^ > 2σ(*F* ^2^)] = 0.038
*wR*(*F* ^2^) = 0.093
*S* = 1.06
1147 reflections
92 parameters
H-atom parameters constrained
Δρ_max_ = 0.86 e Å^−3^
Δρ_min_ = −0.62 e Å^−3^

### 

Data collection: *COLLECT* (Hooft, 1998 ▶); cell refinement: *DENZO* (Otwinowski & Minor, 1997 ▶) and *COLLECT*; data reduction: *DENZO* and *COLLECT*; program(s) used to solve structure: *SHELXS97* (Sheldrick, 2008 ▶); program(s) used to refine structure: *SHELXL97* (Sheldrick, 2008 ▶); molecular graphics: *ORTEP-3* (Farrugia, 1997 ▶) and *DIAMOND* (Brandenburg, 2006 ▶); software used to prepare material for publication: *publCIF* (Westrip, 2010 ▶).

## Supplementary Material

Crystal structure: contains datablocks global, I. DOI: 10.1107/S1600536810003314/hb5318sup1.cif

Structure factors: contains datablocks I. DOI: 10.1107/S1600536810003314/hb5318Isup2.hkl

Additional supplementary materials:  crystallographic information; 3D view; checkCIF report

## Figures and Tables

**Table 1:** Hydrogen-bond geometry (Å, °)

*D*—H⋯*A*	*D*—H	H⋯*A*	*D*⋯*A*	*D*—H⋯*A*
O1—H1⋯N1^i^	0.84	1.85	2.685 (5)	173
C5—H5⋯O2^ii^	0.95	2.39	3.258 (7)	152
C6—H6⋯O2^iii^	0.95	2.47	3.171 (6)	131

Symmetry codes: (i) 
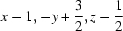
; (ii) 
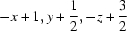
; (iii) 
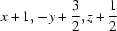
.

**Fig. 1.**
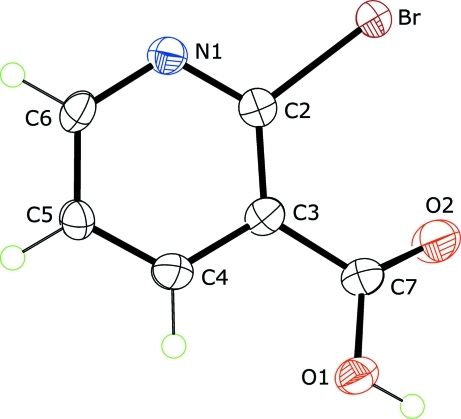
The molecular structure of (I) showing displacement ellipsoids at the 50% probability level.

**Fig. 2.**
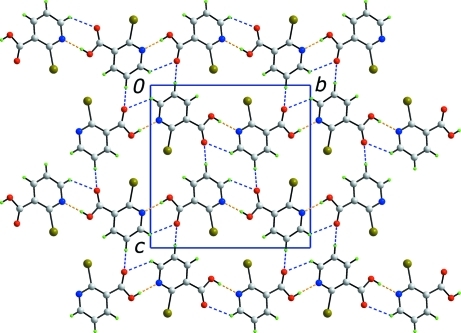
View of the unit cell contents in (I) highlighting the N–H···O hydrogen bonding (orange dashed lines) leading to supramolecular chains, and C–H···O contacts within and between chains (blue dashed lines). Colour code: Br, olive; O, red; N, blue; C, grey; and H, green.

## supplementary crystallographic information

### Comment

The structure of the title compound, (I), was determined in connection with on-going studies of biological activitiess, e.g. anti-mycobacterial activity, of N-heterocyclic compounds (Cunico *et al.* 2006; de Souza, 2005), as we have embarked on complementary systematic structural investigations in order to ascertain supramolecular aggregation patterns (Kaiser *et al.*, 2009).

In the molecular structure of (I), Fig. 1, the carbonyl-O2 atom is approximately syn to the bromide. The carboxylic acid residue is twisted out of the plane of the pyridine ring as seen in the value of the C2/C3/C7/O1 torsion angle of 161.1 (5)°. In the crystal packing, a supramolecular chain with base vector [2 0 1] is formed through the agency of O–H···N hydrogen bonds, Fig. 2 and Table 1. Additional stabilisation to the chains are afforded by *C*–*H*···O_carbonyl_ interactions, Table 1. The chains stack into layers in the ab place and are consolidated in the crystal structure by further *C*–*H*···O_carbonyl_ contacts, Fig. 2 & Table 1. Similar supramolecular chains are found in the crystal structures of nicotinic acid (Wright & King, 1953; Kutoglu & Scheringer, 1983) as well as in 2-chloropyridine-3-carboxylic acid (de Souza *et al.*, 2005).

### Experimental

A mixture of 2-bromo-3-methylpyridine (0.77 g, 4.5 mmol), KMnO_4_ (0.316 g, 2 mmol) and H_2_O (20 ml) was refluxed until the purple colour of the solution disappeared. A second portion of KMnO_4_ (0.316 g) and water (10 ml) were added and the reaction mixture was refluxed again until no purple colour remained. The reaction mixture was concentrated to 10 ml, acidified with concentrated hydrochloric acid, and filtered. The precipitate was washed with cold water and cold diethylether (20 ml). The yield was 0.79 g (60%), m.p. 520–523 K; lit value 522-523 K (Bradlow & van der Werf, 1949). 2-Bromonicotinic acid was recrystallised from EtOH for the crystallographic study. ^1^H NMR [500.00 MHz, DMSO-d_6_] δ: 8.50 (1*H*, dd, *J* = 5.0 and 2.0 Hz, H6), 8.13 (1*H*, dd, *J* = 7.5 and 2.0 Hz, H4), 7.55 (1*H*, dd, *J* = 7.5 and 5.0 Hz, H5), 3.44 (1*H*, s, OH) p.p.m. ^13^C NMR (125.0 MHz, DMSO-d_6_) δ: 166.3, 151.8, 139.1, 138.6, 131.1, 123.2 p.p.m.

### Refinement

The C-bound H atoms were geometrically placed (C–H = 0.95 Å) and refined as riding with U_iso_(H) = 1.2U_eq_(C). The N-bound H atoms were located from a difference map and refined with U_iso_(H) = 1.5U_eq_(N).

### Crystal data

**Table:**

C_6_H_4_BrNO_2_	*F*(000) = 392
*M**_r_* = 202.01	*D*_x_ = 2.062 Mg m^−^^3^
Monoclinic, *P*2_1_/*c*	Mo *K*α radiation, λ = 0.71073 Å
Hall symbol: -P 2ybc	Cell parameters from 24006 reflections
*a* = 3.9286 (3) Å	θ = 2.9–27.5°
*b* = 12.9737 (9) Å	µ = 6.24 mm^−^^1^
*c* = 12.8570 (8) Å	*T* = 120 K
β = 96.695 (4)°	Block, colourless
*V* = 650.83 (8) Å^3^	0.10 × 0.09 × 0.08 mm
*Z* = 4	

### Data collection

**Table:**

Nonius KappaCCD area-detector diffractometer	1147 independent reflections
Radiation source: Enraf Nonius FR591 rotating anode	882 reflections with *I* > 2σ(*I*)
10 cm confocal mirrors	*R*_int_ = 0.070
Detector resolution: 9.091 pixels mm^-1^	θ_max_ = 25.0°, θ_min_ = 3.2°
φ and ω scans	*h* = −4→4
Absorption correction: multi-scan (*SADABS*; Sheldrick, 2007)	*k* = −15→15
*T*_min_ = 0.453, *T*_max_ = 0.607	*l* = −15→13
7699 measured reflections	

### Refinement

**Table:**

Refinement on *F*^2^	Primary atom site location: structure-invariant direct methods
Least-squares matrix: full	Secondary atom site location: difference Fourier map
*R*[*F*^2^ > 2σ(*F*^2^)] = 0.038	Hydrogen site location: inferred from neighbouring sites
*wR*(*F*^2^) = 0.093	H-atom parameters constrained
*S* = 1.06	*w* = 1/[σ^2^(*F*_o_^2^) + (0.0439*P*)^2^ + 1.1585*P*] where *P* = (*F*_o_^2^ + 2*F*_c_^2^)/3
1147 reflections	(Δ/σ)_max_ = 0.001
92 parameters	Δρ_max_ = 0.86 e Å^−^^3^
0 restraints	Δρ_min_ = −0.62 e Å^−^^3^

### Special details

**Table:**

Geometry. All s.u.'s (except the s.u. in the dihedral angle between two l.s. planes) are estimated using the full covariance matrix. The cell s.u.'s are taken into account individually in the estimation of s.u.'s in distances, angles and torsion angles; correlations between s.u.'s in cell parameters are only used when they are defined by crystal symmetry. An approximate (isotropic) treatment of cell s.u.'s is used for estimating s.u.'s involving l.s. planes.
Refinement. Refinement of *F*^2^ against ALL reflections. The weighted *R*-factor wR and goodness of fit *S* are based on *F*^2^, conventional *R*-factors *R* are based on *F*, with *F* set to zero for negative *F*^2^. The threshold expression of *F*^2^ > 2σ(*F*^2^) is used only for calculating *R*-factors(gt) etc. and is not relevant to the choice of reflections for refinement. *R*-factors based on *F*^2^ are statistically about twice as large as those based on *F*, and *R*- factors based on ALL data will be even larger.

### Fractional atomic coordinates and isotropic or equivalent isotropic displacement parameters (Å^2^)

**Table:**

	*x*	*y*	*z*	*U*_iso_*/*U*_eq_	
Br	0.26644 (13)	0.59770 (4)	0.89357 (4)	0.0284 (2)	
O1	−0.1485 (10)	0.8088 (3)	0.6204 (3)	0.0329 (9)	
H1	−0.2563	0.7783	0.5691	0.049*	
O2	0.0225 (11)	0.6488 (3)	0.6647 (3)	0.0484 (12)	
N1	0.5499 (10)	0.7843 (3)	0.9471 (3)	0.0257 (10)	
C2	0.3563 (13)	0.7384 (4)	0.8676 (4)	0.0237 (11)	
C3	0.2287 (12)	0.7899 (4)	0.7756 (4)	0.0246 (11)	
C4	0.3069 (14)	0.8942 (4)	0.7698 (4)	0.0303 (12)	
H4	0.2193	0.9327	0.7098	0.036*	
C5	0.5111 (13)	0.9428 (4)	0.8507 (4)	0.0252 (12)	
H5	0.5688	1.0137	0.8461	0.030*	
C6	0.6276 (13)	0.8854 (4)	0.9378 (4)	0.0268 (12)	
H6	0.7679	0.9179	0.9935	0.032*	
C7	0.0240 (13)	0.7406 (4)	0.6822 (4)	0.0290 (12)	

### Atomic displacement parameters (Å^2^)

**Table:**

	*U*^11^	*U*^22^	*U*^33^	*U*^12^	*U*^13^	*U*^23^
Br	0.0342 (3)	0.0217 (3)	0.0277 (3)	−0.0033 (2)	−0.0029 (2)	0.0016 (2)
O1	0.042 (2)	0.030 (2)	0.025 (2)	0.0023 (18)	−0.0059 (17)	−0.0007 (16)
O2	0.072 (3)	0.027 (2)	0.041 (2)	0.009 (2)	−0.018 (2)	−0.0066 (19)
N1	0.031 (2)	0.024 (2)	0.023 (2)	0.0023 (19)	0.0034 (19)	−0.0008 (19)
C2	0.021 (2)	0.024 (3)	0.026 (3)	0.002 (2)	0.005 (2)	−0.001 (2)
C3	0.023 (3)	0.026 (3)	0.024 (3)	0.005 (2)	0.002 (2)	−0.003 (2)
C4	0.035 (3)	0.025 (3)	0.029 (3)	0.003 (2)	−0.003 (2)	0.003 (2)
C5	0.030 (3)	0.020 (3)	0.026 (3)	−0.001 (2)	0.003 (2)	−0.003 (2)
C6	0.027 (3)	0.028 (3)	0.024 (3)	0.000 (2)	−0.002 (2)	−0.007 (2)
C7	0.028 (3)	0.031 (3)	0.027 (3)	0.003 (2)	0.000 (2)	0.002 (2)

### Geometric parameters (Å, °)

**Table:**

Br—C2	1.897 (5)	C3—C4	1.392 (7)
O1—C7	1.322 (6)	C3—C7	1.507 (7)
O1—H1	0.8400	C4—C5	1.388 (7)
O2—C7	1.213 (6)	C4—H4	0.9500
N1—C2	1.340 (6)	C5—C6	1.378 (7)
N1—C6	1.356 (6)	C5—H5	0.9500
C2—C3	1.400 (7)	C6—H6	0.9500
			
C7—O1—H1	109.5	C3—C4—H4	119.5
C2—N1—C6	118.4 (4)	C6—C5—C4	118.1 (5)
N1—C2—C3	123.3 (5)	C6—C5—H5	121.0
N1—C2—Br	113.2 (3)	C4—C5—H5	121.0
C3—C2—Br	123.5 (4)	N1—C6—C5	122.6 (4)
C4—C3—C2	116.7 (5)	N1—C6—H6	118.7
C4—C3—C7	118.1 (4)	C5—C6—H6	118.7
C2—C3—C7	125.2 (4)	O2—C7—O1	123.7 (5)
C5—C4—C3	120.9 (5)	O2—C7—C3	123.8 (5)
C5—C4—H4	119.5	O1—C7—C3	112.6 (4)
			
C2—C3—C7—O1	161.1 (5)	C2—C3—C7—O2	−20.1 (9)
C4—C3—C7—O1	−20.7 (7)	C4—C3—C7—O2	158.1 (6)

### Hydrogen-bond geometry (Å, °)

**Table:**

*D*—H···*A*	*D*—H	H···*A*	*D*···*A*	*D*—H···*A*
O1—H1···N1^i^	0.84	1.85	2.685 (5)	173
C5—H5···O2^ii^	0.95	2.39	3.258 (7)	152
C6—H6···O2^iii^	0.95	2.47	3.171 (6)	131

Symmetry codes: (i) *x*−1, −*y*+3/2, *z*−1/2; (ii) −*x*+1, *y*+1/2, −*z*+3/2; (iii) *x*+1, −*y*+3/2, *z*+1/2.
